# Supplementary material for: Possible Neuroprotective Effects of l-Carnitine on White-Matter Microstructural Damage and Cognitive Decline in Hemodialysis Patients
Source: Nutrients. 2021 Apr 14;13(4):1292. doi: 10.3390/nu13041292 (PMC8070822; doi:10.3390/nu13041292)
Supplement: Supplementary file 1 [file nutrients-13-01292-s001.pdf]

Supplementary material

Supplementary Table 1. Means and standard deviations of scores on neuropsychological tests in the Japanese subjects by age group

		Age (y)		
		55 to 64	65 to 69	≥70
TMT-A (sec)	μ	32	32.1	47.8
	σ	8.4	6.6	14.3
TMT-B (sec)	μ	76	83.3	112.7
	σ	27.9	25.5	31.7
		Age (y)		
Subset of WMS-R		45 to 54	55 to 64	≥65
Logical memory I (points)	μ	22	19.5	7.5
	σ	7.1	6.8	13.2
Logical memory II (points)	μ	16.8	15.3	13.2
	σ	7	7	6.8
Digit span (points)	μ	13	12.8	12.1
	σ	3.1	3.6	3.6
Visual span (points)	μ	15.9	15.3	15.1
	σ	3.3	3.4	2.6

TMT = Trail making test, WMS-R = Wechsler Memory Scale-Revised, μ = mean score, σ = standard deviation

Supplementary Table 2. Tract of interest analysis for fractional anisotropy among hemodialysis patients with no or short-term L-carnitine treatment, long-term L-carnitine treatment , and healthy controls

	Duration of LCAR treatment		HC	*P* values			
	No or short-term *n* = 7	Long-term *n* = 7	*n* =10	Among 3 groups	LTLC vs NSTLC	NSTLC vs HC	LTLC vs HC
Whole FA	0.429±0.013	0.441±0.033	0.469±0.012	<0.001*	0.644	<0.001	0.151
Latr FA	0.454±0.022	0.467±0.039	0.504±0.019	0.020*	0.660	0.003	0.028
Ratr FA	0.449±0.023	0.459±0.033	0.492±0.025	0.010*	0.791	0.012	0.056
Lcs FA	0.615±0.030	0.635±0.032	0.654±0.015	0.018*	0.338	0.014	0.29
Rcs FA	0.617±0.027	0.633±0.035	0.653±0.010	0.019*	0.611	0.024	0.36
Lcc FA	0.611±0.024	0.641±0.031	0.663±0.022	0.002*	0.094	0.001	0.22
Rcc FA	0.537±0.032	0.556±0.052	0.601±0.033	0.008*	0.635	0.008	0.069
Lch FA				0.696			
Rch FA				0.846			
fm FA	0.667±0.025	0.686±0.030	0.716±0.018	0.002*	0.288	0.001	0.057
fmi FA	0.493±0.022	0.506±0.047	0.533±0.017	0.008*	0.786	0.006	0.366
Lifof FA	0.490±0.012	0.517±0.039	0.548±0.023	<0.001*	0.261	<0.001	0.196
Rifof FA	0.500±0.022	0.515±0.046	0.544±0.026	0.032*	0.701	0.005	0.338

Lilf FA	0.469±0.023	0.488±0.041	0.515±0.019	0.004*	0.534	0.003	0.297
Rilf FA	0.475±0.023	0.487±0.039	0.510±0.027	0.080	0.728	0.075	0.317
Lslf FA	0.480±0.018	0.493±0.033	0.526±0.027	0.006*	0.638	0.007	0.057
Rslf FA	0.492±0.032	0.505±0.040	0.539±0.027	0.018*	0.738	0.02	0.105
Luf FA	0.477±0.029	0.500±0.050	0.536±0.019	0.003*	0.588	0.002	0.229
Ruf FA				0.330			
Lslftemp FA				0.052			
Rslftemp FA				0.311			

One-way analysis of variance with the post hoc Tukey or Games-Howell test was used for comparison. * = Values differed significantly (*p*<0.05, FDR corrected). LCAR = L-carnitine; LTLC = long-term L-carnitine treatment; HC = healthy control; NSTLC = no or short-term L-carnitine treatment; FA = fractional anisotropy; FDR = false detection rate; L(R)atr = left(right) anterior thalamic radiation; L(R)cs = corticospinal tract; L(R)cc = cingulum (cingulate gyrus); L(R)ch = cingulum (hippocampus); fm = forceps major; fmi = forceps minor; L(R)ifof = left(right) inferior fronto-occipital fasciculus; L(R)ilf = left(right) inferior longitudinal fasciculus; L(R)slf = left(right) superior longitudinal fasciculus; L(R)uf = left(right) uncinate fasciculus; L(R)slftemp = left(right) superior longitudinal fasciculus temporal part.

Supplementary Table 3. Tract of interest analysis for axial diffusivity among hemodialysis patients with no or short-term L-carnitine treatment, long-term L-carnitine treatment, and healthy controls

	Duration of LCAR treatment			*P* values			
	No or short-term<br>*n* = 7	Long-term<br>*n* = 7	HC<br>*n* =10	Among 3 groups	LTLC vs NSTLC	NSTLC vs HC	LTLC vs HC
Whole AD	1.239±0.025	1.220±0.045	1.199±0.022	0.049	0.517	0.041	0.355
Latr AD	1.324±0.058	1.293±0.084	1.241±0.038	0.029	0.605	0.026	0.201
Ratr AD	1.301±0.043	1.258±0.103	1.217±0.039	0.017	0.446	0.04	0.42
Lcs AD				0.789			
Rcs AD				0.453			
Lcc AD				0.757			
Rcc AD				0.619			
Lch AD				0.831			
Rch AD				0.542			
fm AD				0.96			
fmi AD				0.208			
Lifof AD				0.081			
Rifof AD				0.227			
Lilf AD				0.206			

Rilf AD				0.199			
Lslf AD	1.252±0.027	1.245±0.064	1.202±0.033	0.023	0.958	0.073	0.13
Rslf AD	1.267±0.039	1.254±0.049	1.212±0.041	0.034	0.835	0.039	0.134
Luf AD				0.079			
Ruf AD				0.11			
Lslftemp AD				0.357			
Rslftemp AD	1.458±0.063	1.439±0.063	1.375±0.039	0.011	0.789	0.013	0.062

One-way analysis of variance with the post hoc Tukey or Games-Howell test was used for comparison. LCAR = L-carnitine; LTLC = long-term L-carnitine treatment; HC = healthy control; NSTLC = no or short-term L-carnitine treatment; AD = axial diffusivity; L(R)atr = left(right) anterior thalamic radiation; L(R)cs = corticospinal tract; L(R)cc = cingulum (cingulate gyrus); L(R)ch = cingulum (hippocampus); fm = forceps major; fmi = forceps minor; L(R)ifof = left(right) inferior fronto-occipital fasciculus; L(R)ilf = left(right) inferior longitudinal fasciculus; L(R)slf = left(right) superior longitudinal fasciculus; L(R)uf = left(right) uncinate fasciculus; L(R)slftemp = left(right) superior longitudinal fasciculus temporal part.

Supplementary Table 4. Tract of interest analysis for radial diffusivity among hemodialysis patients with no or short-term L-carnitine treatment, long-term L-carnitine treatment, and healthy controls

	Duration of LCAR treatment			*P* values			
	No or short-term *n* = 7	Long-term *n* = 7	HC *n* =10	Among 3 groups	LTLC vs NSTLC	NSTLC vs HC	LTLC vs HC
Whole RD	0.622±0.020	0.596±0.060	0.553±0.021	<0.001*	0.558	<0.001	0.225
Latr RD	0.655±0.055	0.622±0.091	0.549±0.030	0.003*	0.696	0.004	0.177
Ratr RD	0.642±0.037	0.615±0.092	0.552±0.043	0.016*	0.666	0.016	0.112
Lcs RD	0.459±0.034	0.434±0.055	0.403±0.020	0.009*	0.582	0.009	0.372
Rcs RD	0.460±0.029	0.442±0.059	0.407±0.009	0.004*	0.75	0.005	0.329
Lcc RD	0.468±0.023	0.439±0.051	0.408±0.036	0.016*	0.355	0.011	0.235
Rcc RD	0.525±0.034	0.504±0.065	0.456±0.044	0.017*	0.707	0.025	0.143
Lch RD				0.741			
Rch RD				0.783			
fm RD	0.433±0.030	0.409±0.038	0.370±0.024	0.002*	0.325	0.001	0.039
fmi RD	0.582±0.029	0.562±0.076	0.519±0.022	0.002*	0.804	0.002	0.356
Lifof RD	0.614±0.022	0.580±0.071	0.522±0.029	<0.001*	0.485	<0.001	0.169
Rifof RD	0.607±0.021	0.576±0.063	0.534±0.039	0.001*	0.47	0.001	0.3
Lilf RD	0.612±0.034	0.580±0.059	0.536±0.026	0.002*	0.475	0.001	0.216

Rilf RD	0.618±0.024	0.589±0.061	0.558±0.043	0.041	0.483	0.034	0.341
Lslf RD	0.576±0.031	0.558±0.064	0.503±0.034	0.002*	0.767	0.001	0.154
Rslf RD	0.565±0.037	0.546±0.060	0.490±0.034	0.005*	0.703	0.006	0.043
Luf RD	0.624±0.049	0.584±0.078	0.520±0.021	0.001*	0.51	0.002	0.156
Ruf RD				0.052			
Lslftemp RD	0.557±0.043	0.496±0.085	0.466±0.047	0.020*	0.167	0.015	0.565
Rslftemp RD				0.089			

One-way analysis of variance with the post hoc Tukey or Games-Howell test was used for comparison. * = Values differed significantly (*p*<0.05, FDR corrected). LCAR = L-carnitine; LTLC = long-term L-carnitine treatment; HC = healthy control; NSTLC = no or short-term L-carnitine treatment; FDR = false detection rate; RD = radial diffusivity; L(R)atr = left(right) anterior thalamic radiation; L(R)cs = corticospinal tract; L(R)cc = cingulum (cingulate gyrus); L(R)ch = cingulum (hippocampus); fm = forceps major; fmi = forceps minor; L(R)ifof = left(right) inferior fronto-occipital fasciculus; L(R)ilf = left(right) inferior longitudinal fasciculus; L(R)slf = left(right) superior longitudinal fasciculus; L(R)uf = left(right) uncinate fasciculus; L(R)slftemp = left(right) superior longitudinal fasciculus temporal part.

Supplementary Table 5. Tract of interest analysis for mean diffusivity among hemodialysis patients with no or short-term L-carnitine treatment, long-term L-carnitine treatment, and healthy controls

	Duration of LCAR treatment		HC	*P* values			
	No or short-term *n* = 7	Long-term *n* = 7	HC *n* =10	Among 3 groups	LTLC vs NSTLC	NSTLC vs HC	LTLC vs HC
Whole MD	0.827±0.018	0.804±0.053	0.768±0.021	<0.001*	0.552	<0.001	0.268
Latr MD	0.878±0.054	0.846±0.088	0.780±0.030	0.004*	0.69	0.005	0.207
Ratr MD	0.862±0.035	0.829±0.095	0.774±0.041	0.021*	0.564	0.019	0.18
Lcs MD	0.758±0.018	0.742±0.056	0.716±0.021	0.004*	0.66	0.053	0.294
Rcs MD	0.767±0.022	0.755±0.064	0.721±0.015	0.046	0.825	0.051	0.173
Lcc MD				0.178			
Rcc MD				0.155			
Lch MD				0.941			
Rch MD				0.628			
fm MD	0.814±0.029	0.803±0.028	0.776±0.021	0.017*	0.718	0.018	0.104
fmi MD	0.841±0.030	0.825±0.068	0.786±0.024	0.007*	0.841	0.005	0.349
Lifof MD	0.870±0.031	0.849±0.063	0.795±0.022	0.001*	0.713	0.001	0.146
Rifof MD	0.873±0.022	0.846±0.047	0.812±0.034	0.008*	0.346	0.006	0.151
Lilf MD	0.847±0.040	0.823±0.049	0.783±0.024	0.028*	0.486	0.006	0.091

Rilf MD	0.861±0.030	0.835±0.056	0.805±0.039	0.044	0.502	0.036	0.338
Lslf MD	0.802±0.029	0.787±0.062	0.736±0.029	0.002*	0.839	0.001	0.173
Rslf MD	0.799±0.028	0.782±0.052	0.731±0.032	0.012*	0.688	0.004	0.031
Luf MD	0.868±0.044	0.835±0.064	0.779±0.026	0.001*	0.53	0.002	0.128
Ruf MD	0.845±0.046	0.795±0.051	0.785±0.030	0.022*	0.084	0.021	0.879
Lslftemp MD	0.816±0.032	0.772±0.067	0.741±0.051	0.027*	0.278	0.02	0.446
Rslftemp MD	0.826±0.050	0.795±0.051	0.760±0.028	0.015*	0.382	0.012	0.228

One-way analysis of variance with the post hoc Tukey or Games-Howell test was used for comparison.* = Values differed significantly (p<0.05, FDR corrected). LCAR = L-carnitine; LTLC = long-term L-carnitine treatment; HC = healthy control; NSTLC = no or short-term L-carnitine treatment; FDR = false detection rate; MD = mean diffusivity; L(R)atr = left(right) anterior thalamic radiation; L(R)cs = corticospinal tract; L(R)cc = cingulum (cingulate gyrus); L(R)ch = cingulum (hippocampus); fm = forceps major; fmi = forceps minor; L(R)ifof = left(right) inferior fronto-occipital fasciculus; L(R)ilf = left(right) inferior longitudinal fasciculus; L(R)slf = left(right) superior longitudinal fasciculus; L(R)uf = left(right) uncinate fasciculus; L(R)slftemp = left(right) superior longitudinal fasciculus temporal part.

Supplementary Table 6. Tract of interest analysis for axial kurtosis among hemodialysis patients with no or short-term L-carnitine treatment, long-term L-carnitine treatment, and healthy controls

	Duration of LCAR treatment			*P* values			
	No or short-term *n* = 7	Long-term *n* = 7	HC *n* =10	Among 3 groups	LTLC vs NSTLC	NSTLC vs HC	LTLC vs HC
Whole AK				0.121			
Latr AK				0.084			
Ratr AK				0.05			
Lcs AK				0.342			
Rcs AK				0.255			
Lcc AK				0.278			
Rcc AK				0.137			
Lch AK				0.401			
Rch AK				0.236			
fm AK				0.331			
fmi AK				0.145			
Lifof AK				0.194			
Rifof AK				0.13			
Lilf AK				0.295			

Rilf AK				0.12			
Lslf AK				0.085			
Rslf AK	0.766±0.042	0.783±0.056	0.830±0.043	0.025	0.779	0.028	0.123
Luf AK				0.864			
Ruf AK				0.467			
Lslftemp AK				0.377			
Rslftemp AK				0.256			

One-way analysis of variance with the post hoc Tukey or Games-Howell test was used for comparison. LCAR = L-carnitine; LTLC = long-term L-carnitine treatment; HC = healthy control; NSTLC = no or short-term L-carnitine treatment; AK = axial kurtosis; L(R)atr = left(right) anterior thalamic radiation; L(R)cs = corticospinal tract; L(R)cc = cingulum (cingulate gyrus); L(R)ch = cingulum (hippocampus); fm = forceps major; fmi = forceps minor; L(R)ifof = left(right) inferior fronto-occipital fasciculus; L(R)ilf = left(right) inferior longitudinal fasciculus; L(R)slf = left(right) superior longitudinal fasciculus; L(R)uf = left(right) uncinate fasciculus; L(R)slftemp = left(right) superior longitudinal fasciculus temporal part.

Supplementary Table 7. Tract of interest analysis for radial kurtosis among hemodialysis patients with no or short-term L-carnitine treatment, long-term L-carnitine treatment, and healthy controls

	Duration of LCAR treatment			*P* values			
	No or short-term<br>*n* = 7	Long-term<br>*n* = 7	HC<br>*n* =10	Among 3 groups	LTLC vs NSTLC	NSTLC vs HC	LTLC vs HC
Whole RK	1.001±0.078	1.049±0.088	1.134±0.068	0.006*	0.481	0.005	0.085
Latr RK				0.115			
Ratr RK	1.041±0.061	1.060±0.072	1.171±0.055	0.003*	0.835	0.001	0.004
Lcs RK				0.053			
Rcs RK				0.619			
Lcc RK	1.526±0.146	1.657±0.172	1.849±0.173	0.002*	0.316	0.002	0.07
Rcc RK	1.244±0.147	1.372±0.207	1.636±0.179	0.001*	0.388	0.001	0.019
Lch RK				0.39			
Rch RK				0.394			
fm RK				0.13			
fmi RK				0.051			
Lifof RK	1.095±0.124	1.174±0.130	1.263±0.118	0.036	0.47	0.03	0.324
Rifof RK	1.106±0.125	1.208±0.135	1.332±0.116	0.014*	0.292	0.004	0.131
Lilf RK	0.975±0.124	1.041±0.121	1.128±0.111	0.046	0.559	0.039	0.309

Rilf RK	1.048±0.118	1.109±0.083	1.201±0.108	0.022	0.528	0.019	0.196
Lslf RK	1.287±0.084	1.362±0.117	1.507±0.094	0.003*	0.345	0.001	0.019
Rslf RK	1.413±0.117	1.497±0.127	1.605±0.143	0.024	0.473	0.02	0.241
Luf RK				0.255			
Ruf RK	1.080±0.118	1.244±0.229	1.332±0.190	0.038	0.246	0.03	0.606
Lslftemp RK				0.127			
Rslftemp RK				0.193			

One-way analysis of variance with the post hoc Tukey or Games-Howell test was used for comparison. * = Values differed significantly ($p<0.05$, FDR corrected). LCAR = L-carnitine; LTLC = long-term L-carnitine treatment; HC = healthy control; NSTLC = no or short-term L-carnitine treatment; FDR = false detection rate; RK = radial kurtosis; L(R)atr = left(right) anterior thalamic radiation; L(R)cs = corticospinal tract; L(R)cc = cingulum (cingulate gyrus); L(R)ch = cingulum (hippocampus); fm = forceps major; fmi = forceps minor; L(R)ifof = left(right) inferior fronto-occipital fasciculus; L(R)ilf = left(right) inferior longitudinal fasciculus; L(R)slf = left(right) superior longitudinal fasciculus; L(R)uf = left(right) uncinate fasciculus; L(R)slftemp = left(right) superior longitudinal fasciculus temporal part.

Supplementary Table 8. Tract of interest analysis for mean kurtosis among hemodialysis patients with no or short-term L-carnitine treatment, long-term L-carnitine treatment, and healthy controls

	Duration of LCAR treatment			*P* values			
	No or short-term *n* = 7	Long-term *n* = 7	HC *n* =10	Among 3 groups	LTLC vs NSTLC	NSTLC vs HC	LTLC vs HC
Whole MK				0.084			
Latr MK				0.73			
Ratr MK				0.509			
Lcs MK				0.826			
Rcs MK				0.813			
Lcc MK				0.061			
Rcc MK	0.737±0.054	0.781±0.049	0.832±0.082	0.026	0.436	0.021	0.285
Lch MK				0.591			
Rch MK				0.572			
fm MK				0.512			
fmi MK				0.211			
Lifof MK				0.322			
Rifof MK	0.757±0.059	0.792±0.026	0.817±0.064	0.042	0.457	0.087	0.633
Lilf MK				0.595			

Rilf MK				0.17			
Lslf MK	0.911±0.084	0.946±0.062	1.011±0.076	0.025	0.656	0.032	0.204
Rslf MK				0.052			
Luf MK				0.412			
Ruf MK				0.206			
Lslftemp MK				0.645			
Rslftemp MK				0.304			

One-way analysis of variance with the post hoc Tukey or Games-Howell test was used for comparison. LCAR = L-carnitine; LTLC = long-term L-carnitine treatment; HC = healthy control; NSTLC = no or short-term L-carnitine treatment; MK = mean kurtosis; L(R)atr = left(right) anterior thalamic radiation; L(R)cs = corticospinal tract; L(R)cc = cingulum (cingulate gyrus); L(R)ch = cingulum (hippocampus); fm = forceps major; fmi = forceps minor; L(R)ifof = left(right) inferior fronto-occipital fasciculus; L(R)ilf = left(right) inferior longitudinal fasciculus; L(R)slf = left(right) superior longitudinal fasciculus; L(R)uf = left(right) uncinate fasciculus; L(R)slftemp = left(right) superior longitudinal fasciculus temporal part.

Supplementary Table 9. Tract of interest analysis for intra-cellular volume fraction among hemodialysis patients with no or short-term L-carnitine treatment, long-term L-carnitine treatment, and healthy controls

	Duration of LCAR treatment			*P* values			
	No or short-term *n* = 7	Long-term *n* = 7	HC *n* =10	Among 3 groups	LTLC vs NSTLC	NSTLC vs HC	LTLC vs HC
Whole ICVF	0.546±0.293	0.553±0.492	0.600±0.208	0.006*	0.923	0.01	0.025
Latr ICVF	0.614±0.031	0.622±0.041	0.670±0.035	0.008*	0.905	0.012	0.033
Ratr ICVF	0.588±0.033	0.602±0.040	0.648±0.033	0.005*	0.746	0.006	0.035
Lcs ICVF	0.733±0.036	0.725±0.051	0.777±0.024	0.015*	0.911	0.057	0.022
Rcs ICVF	0.721±0.034	0.713±0.050	0.778±0.027	0.003*	0.908	0.014	0.005
Lcc ICVF	0.603±0.047	0.618±0.067	0.683±0.034	0.006*	0.846	0.009	0.035
Rcc ICVF	0.559±0.043	0.581±0.073	0.659±0.035	0.001*	0.764	0.001	0.074
Lch ICVF				0.449			
Rch ICVF				0.123			
fm ICVF	0.623±0.028	0.634±0.312	0.688±0.034	0.001*	0.809	0.001	0.005
fmi ICVF	0.525±0.037	0.548±0.072	0.594±0.029	0.006*	0.739	0.005	0.302
Lifof ICVF	0.535±0.045	0.540±0.057	0.601±0.038	0.011*	0.983	0.022	0.034
Rifof ICVF	0.514±0.050	0.521±0.058	0.586±0.038	0.008*	0.964	0.015	0.028
Lilf ICVF	0.513±0.042	0.510±0.048	0.561±0.028	0.020*	0.993	0.049	0.038

Rilf ICVF	0.502±0.052	0.501±0.062	0.570±0.037	0.011*	0.998	0.028	0.024
Lslf ICVF	0.574±0.038	0.580±0.060	0.651±0.039	0.003*	0.969	0.007	0.013
Rslf ICVF	0.577±0.040	0.591±0.067	0.662±0.040	0.004*	0.861	0.006	0.022
Luf ICVF				0.255			
Ruf ICVF	0.526±0.711	0.524±0.053	0.574±0.033	0.036*	0.998	0.173	0.154
Lslftemp ICVF	0.542±0.049	0.549±0.054	0.608±0.034	0.011*	0.953	0.019	0.037
Rslftemp ICVF	0.565±0.040	0.573±0.053	0.633±0.033	0.005*	0.928	0.009	0.021

One-way analysis of variance with the post hoc Tukey or Games-Howell test was used for comparison.* = Values differed significantly (p<0.05, FDR corrected). LCAR = L-carnitine; LTLC = long-term L-carnitine treatment; HC = healthy control; NSTLC = no or short-term L-carnitine treatment; FDR = false detection rate; ICVF = intra-cellular volume fraction; L(R)atr = left(right) anterior thalamic radiation; L(R)cs = corticospinal tract; L(R)cc = cingulum (cingulate gyrus); L(R)ch = cingulum (hippocampus); fm = forceps major; fmi = forceps minor; L(R)ifof = left(right) inferior fronto-occipital fasciculus; L(R)ilf = left(right) inferior longitudinal fasciculus; L(R)slf = left(right) superior longitudinal fasciculus; L(R)uf = left(right) uncinate fasciculus; L(R)slftemp = left(right) superior longitudinal fasciculus temporal part.

Supplementary Table 10. Tract of interest analysis for the orientation dispersion index among hemodialysis patients with no or short-term L-carnitine treatment, long-term L-carnitine treatment, and healthy controls

	Duration of LCAR treatment		HC	*P* values			
	No or short-term<br>*n* = 7	Long-term<br>*n* = 7	HC<br>*n* =10	Among 3 groups	LTLC vs NSTLC	NSTLC vs HC	LTLC vs HC
Whole ODI				0.109			
Latr ODI				0.277			
Ratr ODI				0.934			
Lcs ODI				0.136			
Rcs ODI				0.325			
Lcc ODI				0.39			
Rcc ODI				0.14			
Lch ODI				0.214			
Rch ODI				0.361			
fm ODI				0.349			
fmi ODI				0.919			
Lifof ODI				0.251			
Rifof ODI				0.74			
Lilf ODI				0.501			

Rilf ODI				0.231			
Lslf ODI	0.162±0.008	0.151±0.010	0.153±0.005	0.028	0.102	0.027	0.885
Rslf ODI				0.511			
Luf ODI				0.093			
Ruf ODI				0.501			
Lslftemp ODI				0.239			
Rslftemp ODI				0.123			

One-way analysis of variance with the post hoc Tukey or Games-Howell test was used for comparison. LCAR = L-carnitine; LTLC = long-term L-carnitine treatment; HC = healthy control; NSTLC = no or short-term L-carnitine treatment; ODI = orientation dispersion index; L(R)atr = left(right) anterior thalamic radiation; L(R)cs = corticospinal tract; L(R)cc = cingulum (cingulate gyrus); L(R)ch = cingulum (hippocampus); fm = forceps major; fmi = forceps minor; L(R)ifof = left(right) inferior fronto-occipital fasciculus; L(R)ilf = left(right) inferior longitudinal fasciculus; L(R)slf = left(right) superior longitudinal fasciculus; L(R)uf = left(right) uncinate fasciculus; L(R)slftemp = left(right) superior longitudinal fasciculus temporal part.

Supplementary Table 11. Region of interest analysis for isotropic volume fraction among hemodialysis patients with no or short-term L-carnitine treatment, long-term L-carnitine treatment, and healthy controls

	Duration of LCAR treatment			*P* values			
	No or short-term *n* = 7	Long-term *n* = 7	HC *n* =10	Among 3 groups	LTLC vs NSTLC	NSTLC vs HC	LTLC vs HC
Whole ISO	0.099±0.008	0.086±0.007	0.086±0.010	0.016	0.033	0.023	0.998
Latr ISO	0.154±0.026	0.118±0.017	0.140±0.037	0.025	0.572	0.028	0.235
Ratr ISO				0.257			
Lcs ISO	0.117±0.011	0.103±0.010	0.101±0.015	0.044	0.058	0.08	0.929
Rcs ISO	0.120±0.013	0.106±0.006	0.107±0.020	0.04	0.211	0.133	0.992
Lcc ISO				0.337			
Rcc ISO	0.073±0.011	0.091±0.018	0.073±0.018	0.038	1	0.074	0.073
Lch ISO				0.388			
Rch ISO				0.174			
fm ISO				0.585			
fmi ISO				0.902			
Lifof ISO				0.094			
Rifof ISO				0.148			
Lilf ISO	0.092±0.019	0.074±0.013	0.070±0.012	0.025	0.034	0.057	0.88

Rilf ISO				0.05			
Lslf ISO				0.362			
Rslf ISO				0.642			
Luf ISO				0.051			
Ruf ISO				0.289			
Lslftemp ISO	0.085±0.021	0.064±0.023	0.051±0.026	0.038	0.032	0.185	0.502
Rslftemp ISO				0.406			

One-way analysis of variance with the post hoc Tukey or Games-Howell test was used for comparison. LCAR = L-carnitine; LTLC = long-term L-carnitine treatment; HC = healthy control; NSTLC = no or short-term L-carnitine treatment; ISO = isotropic volume fraction; L(R)atr = left(right) anterior thalamic radiation; L(R)cs = corticospinal tract; L(R)cc = cingulum (cingulate gyrus); L(R)ch = cingulum (hippocampus); fm = forceps major; fmi = forceps minor; L(R)ifof = left(right) inferior fronto-occipital fasciculus; L(R)ilf = left(right) inferior longitudinal fasciculus; L(R)slf = left(right) superior longitudinal fasciculus; L(R)uf = left(right) uncinate fasciculus; L(R)slftemp = left(right) superior longitudinal fasciculus temporal part.
